# Neuroblastoma is associated with alterations in gut microbiome composition subsequent to maternal microbial seeding

**DOI:** 10.1016/j.ebiom.2023.104917

**Published:** 2023-12-16

**Authors:** Mireia Valles-Colomer, Paolo Manghi, Fabio Cumbo, Giulia Masetti, Federica Armanini, Francesco Asnicar, Aitor Blanco-Miguez, Federica Pinto, Michal Punčochář, Alberto Garaventa, Loredana Amoroso, Mirco Ponzoni, Maria Valeria Corrias, Nicola Segata

**Affiliations:** a Department CIBIO, University of Trento, Trento, Italy; b MELIS Department, Pompeu Fabra University, Barcelona, Spain; c Oncology Unit, IRCCS Istituto Giannina Gaslini, Genoa, Italy; d Laboratory of Experimental Therapies in Oncology, IRCCS Istituto Giannina Gaslini, Genoa, Italy; e Department of Experimental Oncology, IEO European Institute of Oncology IRCCS, Milan, Italy

**Keywords:** Neuroblastoma, Paediatric cancer, Gut microbiome, Metagenomics, Mother-infant transmission

## Abstract

**Background:**

Neuroblastoma is the most frequent extracranial solid tumour in children, accounting for ∼15% of deaths due to cancer in childhood. The most common clinical presentation are abdominal tumours. An altered gut microbiome composition has been linked to multiple cancer types, and reported in murine models of neuroblastoma. Whether children with neuroblastoma display alterations in gut microbiome composition remains unexplored.

**Methods:**

We assessed gut microbiome composition by shotgun metagenomic profiling in an observational cross-sectional study on 288 individuals, consisting of patients with a diagnosis of neuroblastoma at disease onset (N = 63), healthy controls matching the patients on the main covariates of microbiome composition (N = 94), healthy siblings of the patients (N = 13), mothers of patients (N = 59), and mothers of the controls (N = 59). We examined taxonomic and functional microbiome composition and mother-infant strain transmission patterns.

**Findings:**

Patients with neuroblastoma displayed alterations in gut microbiome composition characterised by reduced microbiome richness, decreased relative abundances of 18 species (including *Phocaeicola dorei* and *Bifidobacterium bifidum*), enriched protein fermentation and reduced carbohydrate fermentation potential. Using machine learning, we could successfully discriminate patients from controls (AUC = 82%). Healthy siblings did not display such alterations but resembled the healthy control group. No significant differences in maternal microbiome composition nor mother-to-offspring transmission were detected.

**Interpretation:**

Patients with neuroblastoma display alterations in taxonomic and functional gut microbiome composition, which cannot be traced to differential maternal seeding. Follow-up research should include investigating potential causal links.

**Funding:**

Italian Ministry of Health Ricerca Corrente and Ricerca Finalizzata 5 per mille (to MPonzoni); Fondazione Italiana Neuroblastoma (to MPonzoni); 10.13039/501100000781European Research Council (ERC-StG project MetaPG-716575 and ERC-CoG microTOUCH-101045015 to NS); the European H2020 program ONCOBIOME-825410 project (to NS); the National Cancer Institute of the National Institutes of Health 1U01CA230551 (to NS); the Premio Internazionale Lombardia e Ricerca 2019 (to NS); the MIUR Progetti di Ricerca di Rilevante Interesse Nazionale (PRIN) Bando 2017 Grant 2017J3E2W2 (to NS); EMBO ALTF 593-2020 and Knowledge Generation Project from the Spanish Ministry of Science and Innovation (PID2022-139328OA-I00) (to MV-C).

**Research in context**

**Evidence before this study**

Neuroblastoma is one of the most common paediatric solid cancers. While multiple types of cancer have been associated with an altered gut microbiome composition and neuroblastoma was found linked to microbiome alterations in murine models, gut microbiome composition in patients with neuroblastoma remains unexplored.

**Added value of this study**

Assessing gut microbiome composition by shotgun metagenomic profiling in patients with neuroblastoma at disease onset, we find that this paediatric cancer is associated with microbiome alterations characterised by a reduced species richness and differential species and functional pathway abundances. We do not detect such alterations in healthy siblings, and the mothers of the patients and of the controls do not display significant differences in microbiome composition. The fact that mother-offspring strain transmission was similar between patients with neuroblastoma and healthy controls suggests that maternal seeding is not involved in the pathogenesis of neuroblastoma.

**Implications of all the available evidence**

Neuroblastoma is associated with an altered gut microbiome composition at both taxonomic and functional levels. Identifying whether the gut microbiome plays a causative role in neuroblastoma development or response to therapy could open new venues for development of complementary microbiome-targeted therapeutic approaches.

## Introduction

Neuroblastoma is among the most common malignant neoplasms affecting children and the most frequent extracranial solid tumour, accounting for approximately 8% cases and 15% of deaths due to cancer in childhood.^1^^,^^2^ Its onset is typically at very early ages, with an average age of 18 months at diagnosis and 90% of cases being diagnosed before the age of 5.^3^^,^^4^ Although its incidence is much lower in adolescents and adults, their prognosis is much less favourable.^5^ Neuroblastoma develops from immature nerve cells and although it can arise in any body locations with sympathetic nervous tissue, the most common primary tumour location (65% of cases) is in the abdomen.^6^^,^^7^ It remains a very enigmatic paediatric tumour, displaying high variability in clinical behaviour.^6^ It displays a slight male predominance (ratio 1.2:1)^8^ and some genetic features of the disease have been identified, with a family history being noted for 1–2% of the children diagnosed.^9^ Environmental factors including exposure to drugs, chemicals, and viral infections have also been postulated, but no clear etiologic factors have yet been identified.^6^

The composition of the gut microbiome (the collection of bacteria, archaea, viruses, and eukaryotes colonising the gastrointestinal tract) has been found associated–albeit without causal proof in many cases–with multiple cancer types, including colon, gastric, hepatobiliary, skin, and breast cancer, and it has been proposed to affect tumorigenesis and tumour growth.^10^ Indeed, microbial diagnostic signatures have been identified across cohorts,^11^ and multiple links between microbiome composition and response to therapy are being described.^12^ In murine models of neuroblastoma, increased intestinal barrier permeability, pro-inflammatory cytokines, and alterations in microbiome composition in ileal and faecal contents have been reported.^13^^,^^14^ In addition, intracellular microorganisms in the tumour tissue (assessed with RNA-seq) were found to hold some predictive power for survival in 120 patients with neuroblastoma.^15^ However, to our knowledge alterations in gut microbiome composition in patients with neuroblastoma have not yet been investigated. Here, as the most frequent primary location of tumours in neuroblastoma is in the abdomen and gut microbiome composition has been found altered in numerous (gastrointestinal (GI) and extra-GI) cancers and in murine models of neuroblastoma, we hypothesised that members of the gut microbiome could be involved in the pathogenesis of neuroblastoma.

Microbiome colonisation of the human gastrointestinal tract sets off by maternal transmission during birth,^16^ followed by microbial succession processes that conduct to stabilisation into a more adult-like microbiome (characterised by higher microbial richness and load^17^) around three years of age^18^^,^^19^–past the average age of diagnosis of neuroblastoma. Maternal seeding is disrupted in infants born by C-section,^20^^,^^21^ and while after three years of age children share similar rates of strains with their mothers regardless of their mode of delivery,^22^ missing specific bacteria at key neurodevelopmental windows might have long-lasting effects on children's health.^23^ The gut microbiome is also extensively transmitted among individuals in close proximity and along social networks,^24^, ^25^, ^26^ which raises the possibility that microbiome-associated non-communicable diseases can be (at least partially) communicable.^22^^,^^27^

Here, we assessed gut microbiome composition by shotgun metagenomic profiling in patients with neuroblastoma at disease onset as compared to 1) a group of healthy controls balanced on the main covariates of microbiome composition (age, sex, stool consistency, mode of delivery [vaginal or C-section], and mode of feeding [breastmilk or formula]) (N = 94) and 2) healthy siblings of the patients with neuroblastoma. To examine potential mother-to-infant strain transmission of the microbial signatures of neuroblastoma identified, we also enrolled a group of mothers of the patients and mothers of healthy controls. We found microbiome taxonomic and functional differences in patients with neuroblastoma that in our study could not be traced to differential maternal microbiome composition nor mother-to-infant transmission, thus such alterations might more likely arise after or independently from the initial maternal seeding of the gut microbiome.

## Methods

### Description of the cohort

A cohort of 63 individuals with a diagnosis of neuroblastoma at disease onset (0–27 years old, median = 2.7 y, IQR = [0.9, 5.9]y), 59 of their mothers (19–52 years old), and 13 of their siblings (0–22 years old), together with 94 healthy children (0–13 years old), and 59 of their mothers (27–49 years old) were enrolled at the IRCCS Istituto Giannina Gaslini in Genoa (Italy) between May 2019 and June 2022 (Liguria Regional Ethics Committee, Italy, 006/2019). Being the first study on the gut microbiome in neuroblastoma in humans, sample size determination could not be calculated a priori for this study. Inclusion criteria were diagnosis of neuroblastoma and not having yet started therapy for the NB group, being the mother or sibling of a patient diagnosed with neuroblastoma for the NB mothers and NB siblings groups, and to not have nor have had an oncological disease for the two HC and HC mothers groups, in addition to being willing to participate and provide written informed consent for all groups. Exclusion criteria included having acute or chronic gastroenteric disease, metabolic disease, cystic fibrosis, and autoimmune disorders. A questionnaire on relevant covariates for microbiota composition (age, sex, delivery and feeding modes, stool consistency) was filled by all subjects enrolled in the study or the parents of underaged participants. Stool samples were collected in DNA/RNA shield faecal collection tubes (Zymo Research) and stored at −80 °C until DNA extraction. DNA extraction was performed with the DNeasy PowerSoil Pro Kit (QIAGEN, Germany) according to the manufacturer's procedures. DNA concentration was measured using the NanoDrop spectrophotometer (Thermo Fisher scientific) and stored at −20 °C. Sequencing libraries were prepared using the NexteraXT DNA Library Preparation Kit (Illumina, California, USA), following the manufacturer's guidelines. Sequencing was performed on the Illumina NovaSeq 6000 platform following manufacturer's protocols.

### Metagenome pre-processing and quality control

Samples were pre-processed using the pipeline described in https://github.com/SegataLab/preprocessing. Shortly, metagenomic reads were quality controlled and reads of low quality (quality score < Q20), fragmented short reads (<75 bp), and reads with >2 ambiguous nucleotides were removed with Trim Galore (v0.6.6). Contaminant and host DNA was identified with Bowtie2 (v2.3.4.3)^28^ using the–sensitive–local parameter, allowing confident removal of the Instrument's spike-ins and human-associated reads (hg19 human genome release). Read statistics of all samples (number of reads, number of bases, minimum, median, and maximum read length per sample) are detailed in Table S1. Metagenomes with ≥5 M reads were included in the analysis (N = 288, median = 50 M reads/sample, IQR = [24, 267]).

### Gut microbiome species-level profiling

Species-level profiling was performed with MetaPhlAn 4.0.3^29^ with default parameters and the mpa_vJan21_CHOCOPhlAnSGB_202103 database (available at http://cmprod1.cibio.unitn.it/biobakery4/metaphlan_databases/). A total of 2050 species-level genome bins (SGBs) were detected, including 603 uSGBs (i.e. species with no sequenced cultured representatives) and 1447 kSGBs (species including reference genomes from cultured microorganisms).

### Gut microbiome species-level beta diversity and ordination

Because of the compositional structure of microbiome data, species-level abundance matrices obtained in MetaPhlAn were centred log ratio-transformed using the codaSeq.clr function in the CoDaSeq R package (v0.99.6),^30^ using the minimum proportional abundance detected for each species for the imputation of zeros. A PCoA on Aitchison distance (Euclidean distance between samples after centred log-ratio transformation) was produced with the ordinate and plot_ordination functions in phyloseq (v1.28.0).^31^ Species-level richness (Observed) and diversity (Simpson's) were determined using the estimate_richness function in phyloseq on the untransformed species-level abundance MetaPhlAn matrix, and evenness (Pielou) was calculated as the Shannon's diversity divided by the logarithm of the Observed richness.^32^

### Building a machine learning classifier

To classify the individuals in different groups based on their gut microbiome composition, two random forest classifiers were built with MetAML^33^ (which implements the scikit-learn python library Random Forest (RF)^34^), similarly to the method used in.^11^^,^^12^^,^^35^ The first random forest model was built to classify samples from the HC and NB groups, while the second classifier classified samples from the NB siblings from the NB group while using the HC group in the training phase. Species relative abundances were fed into the classifiers, with 10% of the species being randomly-selected as input to each tree. 1000 estimator trees were built with a minimum of 5 samples per leaf, no fixed depth, and the Gini index as impurity criterion. To assess the performance of the random forest models, 10-fold and 5-fold stratified cross validation respectively (to balance the number of samples of the two groups and account for the different number of samples in each classifier) randomised over 10 iterations was performed. Next, a Receiver Operating Characteristic (ROC) curve was plotted using a linear interpolation, and the Area Under the Curve (AUC), an unbiased estimator, was computed as the average of all the tests performed. 95% confidence intervals of the AUC were computed under the assumption of t-distribution around each set of predicted points. Feature rankings were extracted by the MetAML software from each training fold, and to avoid overfitting, the importance of each species was obtained by averaging the scores obtained in all the rounds of training.^11^

### Functional profiling of gut microbiome composition

Profiling of the functional potential of the microbiome was performed with HUMAnN 3.6^36^ with default parameters and the v30_CHOCOPhlAn_201901 database. Uniref90 IDs were collapsed to KEGG Orthology (KO) terms with the “humann_regroup_table” function provided within HUMAnN3. Gut-brain module (v1.0)^37^ and gut metabolic module (v1.07)^38^ abundances were calculated with omixer-rpmR (v0.3.3).^39^

### Strain-level profiling of gut microbiome composition

Strain profiling was performed with StrainPhlAn 4^29^ using the mpa_vJan21_CHOCOPhlAnSGB_202103 database as in.^40^ Shortly, parameters “–sample_with_n_markers 20–secondary_samples_with_n_markers 20–sample_with_n_markers_after_filt 10–marker_in_n_samples 50–mutation_rates” were used, and to reduce noise only markers present in 50% of the samples and samples with at least 20 markers prior to filtering and 10 markers after filtering were kept. To more confidently define strain identity, independent samples from the curatedMetagenomicData (cMD) resource (v.3.15)^41^ were included. These consisted of 4443 human gut metagenomic samples from 962 individuals older than 6 years from populations defined as ‘Westernised’ in cMD that were sampled longitudinally, obtained from 18 datasets (see^40^). For each subject and each SGB, two samples being at most 6 months apart were selected. When more than two timepoints close in time were available, we selected the pair that maximised the lower estimated coverage of the SGB among the two samples, that is, maximised their chance to pass the filtering steps in StrainPhlAn. In case of ties, we took those with higher coverage. Coverage of an SGB in a sample was estimated as [sample sequencing depth] × [relative abundance of the SGB]/[estimated genome length], with estimated genome length being extracted from the MetaPhlAn database. For kSGBs coverage was determined using only the genome lengths of the reference genomes in the kSGB, whereas for uSGBs 7% was added to the average genome length [estimated to be the average difference between the genome sizes of reference genomes and metagenome-assembed genomes (MAGs) within the same SGB]. Samples were defined as primary in StrainPhlAn (i.e. those that are used to select markers) if they had an estimated coverage of at least 2X for a given SGB genome, and as secondary otherwise (and thus added only after the markers were selected using the primary samples only). Next, following the method in,^40^ strains of putative food origin were identified and excluded. To this end, we included 216 MAGs assembled in food samples in 19 SGBs and used them in StrainPhlAn with the–secondary_references parameters. Samples with StrainPhlAn mutation rates below 0.0015 to any food MAG were discarded following the same procedure as in.^22^ In total, 799 SGBs profiled at the strain level were included in the analyses.

### Assessment of mother-to-offspring transmission

To detect strain sharing events, we used the strain identity thresholds in the *strain_transmission.py* script in StrainPhlAn 4 (–threshold argument), https://github.com/biobakery/MetaPhlAn/blob/master/metaphlan/utils/strain_transmission.py”, following the procedure in^22^ and the longitudinal datasets in.^40^ Shortly, pairs of strains with pairwise phylogenetic distance below the strain identity threshold were defined as strain sharing events. Person-to-person strain sharing rates were calculated as the number of strains shared between two individuals divided by the number of shared SGBs profiled by StrainPhlAn (N shared strains/N shared SGBs) as a normalisation method. For a robust calculation, person-to-person strain sharing rates were only assessed when at least 5 SGBs were shared between two individuals. Species transmissibility was defined as the number of strain sharing events detected for an SGB divided by the total potential number of strain sharing events based on the presence of a strain-level profile by StrainPhlAn 4 as a normalisation method. For a robust calculation, SGB transmissibility was only assessed on SGBs with at least five potential strain sharing events.

### Statistical analysis

Statistical analyses and graphical representations were performed in R using packages vegan (version 2.5–7),^42^ phyloseq (v1.28.0),^31^ ggplot2 (v3.3.3),^43^ and ggpubr (v0.4.0).^44^ The association between metadata variables and microbiota community variation was assessed by distance-based redundancy analysis (dbRDA) on the species-level Aitchison distance matrix with the *capscale* function in the vegan R package. The cumulative contribution of metadata variables was determined by forward model selection on dbRDA with the ordiR2step function in vegan, with variables that showed a significant contribution to microbiota community variation in the previous step. Differential abundance analyses were performed on species with an average relative abundance greater than 0.5% in non-transformed data and present in at least 20 samples in the groups being compared (N = 43) and in functions with an average relative abundance greater than 0.01% in non-transformed data and present in at least 20 samples in the groups being compared [N = 150 MetaCyc pathways, N = 86 Gut Metabolic Modules (GMMs), N = 35 Gut-Brain Modules (GBMs)]. Differences between two groups were assessed with Wilcoxon rank-sum tests, as no correction for microbiome covariates was required with the NB and HC groups being balanced. A positive effect size (r) denotes higher values in the control group, while a negative one denotes lower values in the case group. For more than two groups, the Kruskal–Wallis test with post hoc Dunn tests was used. Correlations were assessed with Spearman's tests. All tests were two-sided except where otherwise specified. Correction for multiple testing (Benjamini–Hochberg procedure, Padj) was applied when appropriate and significance was defined at Padj < 0.05.

### Ethical compliance

All study procedures are compliant with all relevant ethical regulations. The procedures were performed in compliance with the Declaration of Helsinki. Ethical approval was granted by the Liguria Regional Ethics Committee, Italy (006/2019). Written informed consent was obtained from all adult participants, and from parents of underaged participants.

### Role of funders

The funders did not play any role in study design, data collection, data analyses, interpretation, or writing of the manuscript.

## Results

### A cohort of individuals with neuroblastoma, healthy siblings and controls, and their mothers

**Fig. 1:**
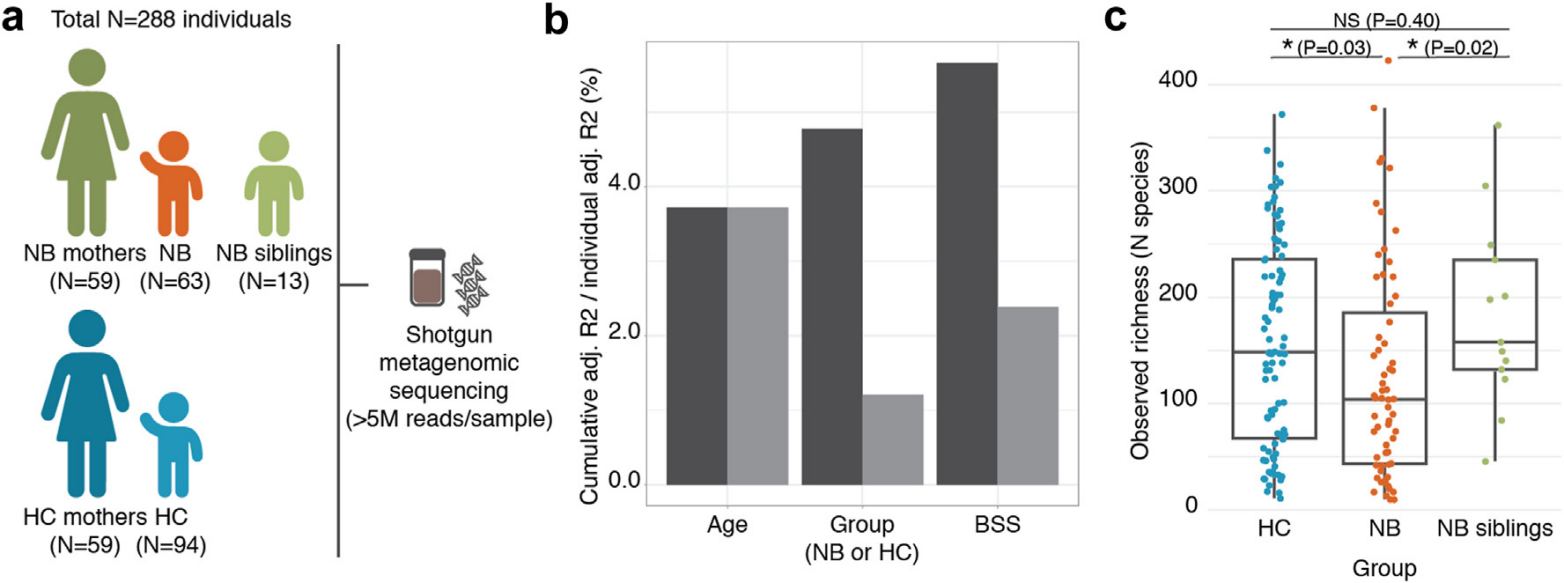
**Study design and overview of the cohort**. **a**. A total of 288 individuals were enrolled in the study, including individuals with a diagnosis of neuroblastoma at disease onset, their healthy siblings, their mothers, a group of healthy individuals balanced to the patient group, and their mothers (Tables S1 and S2). Inclusion and exclusion criteria are detailed in the Methods section. Gut microbiome composition was assessed by shotgun metagenomic sequencing on stool samples. **b**. Cumulative effect sizes of significant covariates (P < 0.05) on species-level microbiome community variation (dark grey, left bars; stepwise dbRDA on Aitchison distance) compared to individual effect sizes assuming covariate independence (light grey, right bars; dbRDA on Aitchison distance) in the NB (N = 63) and HC (N = 94) groups (Table S3). Age, sex, group, Bristol Stool Scale (BSS), mode of delivery, and mode of feeding were tested as potential microbiome covariates. **c**. Observed species richness distribution in the HC (N = 94), NB (N = 63), and NB siblings (N = 13) groups. Wilcoxon rank-sum tests (∗P < 0.05, NS P ≥ 0.05; Table S4).

A total of 288 individuals were enrolled at the IRCCS Istituto Giannina Gaslini (Genoa, Italy) between 2019 and 2022, consisting of 63 patients with a diagnosis of neuroblastoma at disease onset (*NB group*), 94 healthy individuals (*HC group*), 13 healthy siblings of patients (*NB siblings group*), 59 mothers of patients (*NB mothers group*), and 59 mothers of the healthy controls (*HC mothers group*) (Fig. 1a; Table S1). The NB and HC groups were balanced for the main covariates of gut microbiome composition that have been described for either infants^18^ or adults^45^ [age, sex, BSS (Bristol Stool Scale, a measure of stool consistency that serves as a proxy for intestinal colon transit time),^46^ mode of delivery (vaginal or C-section), and mode of feeding (breastmilk or formula)] (defined as no significant differences detected for each variable between the two groups; Table 1, Table S2). As patient samples were collected at disease onset, neuroblastoma therapy did not likely constitute a confounding effect. The groups of mothers (NB mothers, HC mothers) were balanced as well on the available covariates of relevance for microbiome composition (Table 1, Table S2). Intake of other medication that could potentially modify microbiome composition was also very limited and balanced across groups (antibiotics: N = 2 in the HC group, N = 3 in the NB group, N = 1 in the HC mothers group, N = 1 in the NB mothers group) (Table S1). Inclusion and exclusion criteria for all groups are detailed in the Methods section. Shotgun metagenomics was performed on stool samples at a minimum of 5 M reads/sample after quality control (median = 50 M reads/sample, IQR = [24, 267] reads/sample).

**Table 1:** Distribution of covariates of gut microbiome composition available in the HC and HC groups.

Variable	HC group	NB group	P
Age (years)	2.95 [0.85, 6.57]	2.7 [0.9, 5.9]	0.73
Sex (female/male)	47/47	27/36	0.47
BSS (score)	4 [3, 6]	3 [2.5, 6]	0.12
Mode of delivery (vaginal/C-section)	58/36	42/21	0.64
Feeding mode (breastmilk/formula)	70/24	48/15	0.96

Variable	HC mothers group	NB mothers group	P
Age (years)	36.80 [33.55, 42.30]	36.70 [33.00, 41.50]	0.75
BSS (score)	3 [3, 4]	3 [3, 4]	0.54

For numerical variables, the median and interquartile range are shown as the data did not display a normal distribution (Shapiro–Wilk test, P < 0.05). For categorical variables, the number of samples in each group is shown. The two groups were compared with Wilcoxon rank-sum tests for numerical variables and with Chi2 tests for categorical variables (Table S2).

### Gut microbiome taxonomic composition is altered in patients with neuroblastoma

Species-level gut microbiome composition differed moderately but significantly between patients with neuroblastoma and healthy controls (Fig. S1; dbRDA, adjusted R2 = 1.2%, P = 4.6e-03). Of the microbiome covariates available, only participant's age and BSS explained a larger proportion of interindividual variation in microbiome composition than the disease (dbRDA, adj. R2 = 3.7%, Padj = 3.5e-03 and adj. R2 = 2.4%, Padj = 3.5e-03; Table S3). Indeed, in a cumulative multivariate model, neuroblastoma provided additional contribution to interindividual microbiota variation beyond participant's age, followed by BSS (Fig. 1b; stepwise dbRDA, total adj. R2 = 5.7%, Padj = 2e-03; Table S3). In contrast, sex, mode of delivery, and mode of feeding were not significant covariates in this cohort (Padj ≥ 0.05; Table S3).

**Fig. 2:**
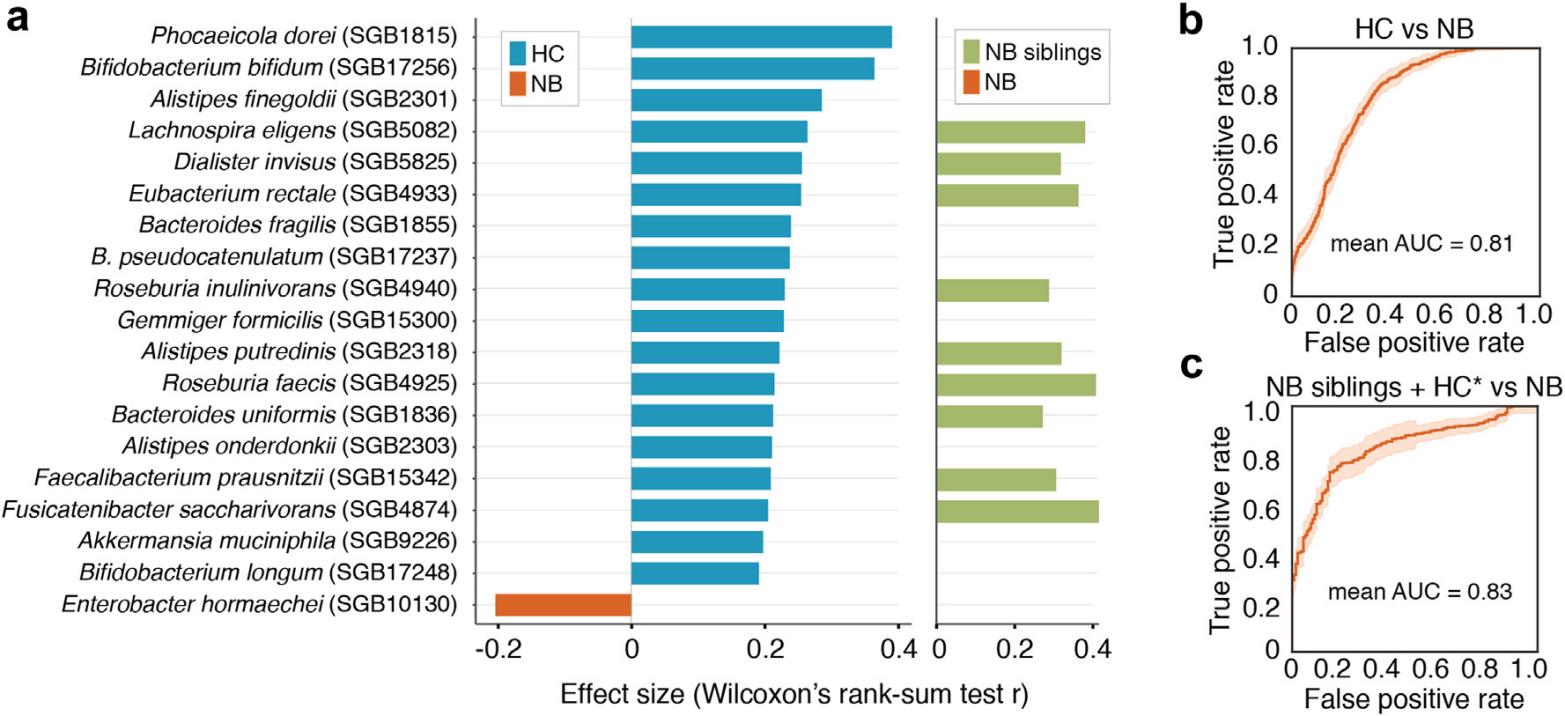
**Microbiome taxonomic composition is altered in patients with neuroblastoma**. **a**. Species displaying significant differences in relative abundances between the HC and NB groups (Wilcoxon rank-sum tests; Padj < 0.05, Table S5) and assessment of differences in relative abundances of NB-associated species in the NB siblings group (Wilcoxon rank-sum tests; Padj < 0.05, Table S6). A positive effect size corresponds to increased relative abundance in the HC group, while a negative one corresponds to increased relative abundance in the NB group. b, c. Receiver Operating Characteristics (ROC) curve of the performance of a Random forest model classifying samples from (**b**) the NB and HC groups, and (**c**) NB and NB siblings (including the HC in the training phase only) based on their based on species-level gut microbiome composition in 10 iterations of a 10- and 5-fold cross-validation respectively. The median Area under the Roc curve (AUC), an indicator of the model performance, is reported on each plot (Methods).

We found the microbiome of patients with neuroblastoma to display reduced species richness (the number of species detected in each sample, i.e. Observed richness) (Fig. 1c; Observed richness, Wilcoxon rank-sum test, r = −0.17, P = 0.03; no significant differences in potentially-confounding sequencing depth detected: Wilcoxon rank-sum test, r = −0.07, P = 0.53). While no significant differences in diversity nor evenness were detected (Simpson's index, P = 0.11; Pielou, P = 0.23), these were also slightly lower in patients (Fig. S2; Table S4). These results were replicated in the healthy siblings of the patients, which also displayed increased richness, diversity, and evenness than the NB group (P < 0.05), together with non-significant differences in richness and diversity when compared to the healthy controls (P ≥ 0.05) (Fig. 1c; Fig. S2; Table S4). At the level of single microbiome members, we identified 18 species with significantly lower relative abundances in patients with neuroblastoma, while only one was enriched in the patient group (Fig. 2a; Wilcoxon rank-sum tests, Padj < 0.05; Table S5). The strongest associations (r > |0.3|) were found for *Phocaeicola dorei* (r = 0.39, Padj = 4.4e-05) (a species previously reported to reduce microbial LPS production^47^), and *Bifidobacterium bifidum* (r = 0.36, Padj = 1.1e-04), both depleted in patients. *B. bifidum* has been granted Qualified Presumption of Safety by the European Food Safety Authority to be used as a probiotic for the prevention and treatment of intestinal diseases and immunological disorders^48^ and this species is typically abundant in breastfed infants as it efficiently catabolises human milk oligosaccharides [HMOs]. *B. bifidum* strains were also reported to work synergically with immune checkpoint inhibitors to reduce tumour burden in murine models of lung cancer.^49^ Two other *Bifidobacterium* species were also found reduced in patients (*B. pseudocatenulatum, B. longum*), together with the health-associated butyrate-producing bacteria *Roseburia inulinivorans, R. faecis, Faecalibacterium prausnitzii,* and *Eubacterium rectale* (Fig. 2a; Table S5). In contrast, the species with increased relative abundances in patients, *Enterobacter hormaechei*, is regarded as mostly pathogenic.^50^

The differences in overall microbiome composition between patients with neuroblastoma and controls were confirmed using a machine learning approach. A random forest classifier (cross-validation with nested model selection, Methods; Fig. S3) was able to discriminate the samples of the two groups based on their species-level microbiome composition with a Area Under the Curve (AUC) = 81% (Fig. 2b). These differences were successfully validated in the NB siblings group when using the HC group only in the training set to discriminate the NB group (AUC = 83%; Fig. 2b). In the random forest algorithm, the percentage of tree estimators in which a given species is used for the final prediction can be used to infer the most important discriminatory features among groups.^11^ Here, the species with the highest importance rankings (Methods) in both models were *B. bifidum* and *P. dorei* (Table S6), the two species found most strongly negatively-associated with neuroblastoma. Next, we assessed whether the microbiome compositional differences identified were also detected in the healthy siblings of the patients with neuroblastoma. Of the 19 neuroblastoma-associated species, 9 were successfully validated, including *Fusicatenibacter saccharinorans, R. inulinivorans, R. faecis, F. prausnitzii, E. rectale, L. eligens, Dialister invisus, Alisipes putredinis,* and *Bacteroides uniformis* (Fig. 2a; Wilcoxon rank-sum tests, Padj < 0.05; Table S7).

### Gut microbiome composition in patients with neuroblastoma is enriched in protein fermentation and reduced in carbohydrate degradation potential

**Fig. 3:**
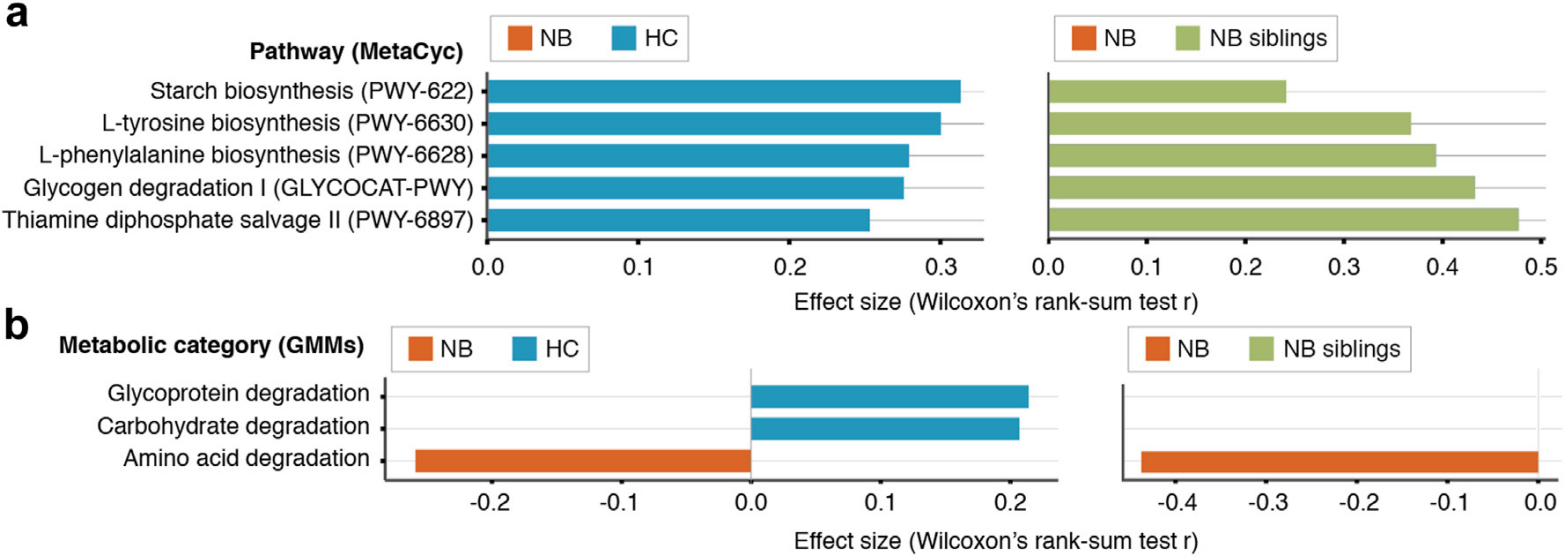
**Microbiome functional composition is altered in patients with neuroblastoma**. **a**. Metabolic pathways (MetaCyc, Methods) displaying significant differences in relative abundances between the HC and NB groups (Wilcoxon rank-sum tests; Padj < 0.05, Table S8) and assessment of differences in relative abundances of NB-associated pathways in the NB siblings group (Wilcoxon rank-sum tests; Padj < 0.05, Table S9). A positive effect size corresponds to increased relative abundance in the HC or NB siblings group (blue and green, respectively), while a negative one corresponds to increased relative abundance in the NB group (orange). **b**. Metabolic categories (Gut Metabolic Modules, Methods) displaying significant differences in relative abundances between the HC and NB groups (Wilcoxon rank-sum tests; Padj < 0.05, Table S12) and assessment of differences in relative abundances of NB-associated pathways in the NB siblings group (Wilcoxon rank-sum tests; Padj < 0.05, Table S13). A positive effect size corresponds to increased relative abundance in the HC or NB siblings group (blue and green, respectively), while a negative one corresponds to increased relative abundance in the NB group (orange).

The gut microbiomes of patients with neuroblastoma and healthy controls also differed significantly on their functional composition (Fig. S4; dbRDA, adjusted R2 = 2.4%, P = 1e-03). Specifically, we found that five MetaCyc metabolic pathways (Methods) were significantly depleted in the NB group (Fig. 3a, Wilcoxon rank-sum tests; Padj < 0.05, Table S8). These included two carbohydrate metabolism pathways: starch biosynthesis from fructose (r = 0.31, Padj = 0.01) and glycogen degradation to glucose 6-phosphate (r = 0.28, Padj = 0.02); two pathways for synthesis of the aromatic amino acids l-tyrosine (r = 0.30, Padj = 0.01) and l-phenylalanine (r = 0.28, Padj = 0.02); and a pathway for synthesis of the vitamin B1 (thiamine diphosphate) (r = 0.25, Padj = 0.04). While tyrosine can also be synthesised by humans by hydroxylation of phenylalanine, both phenylalanine and vitamin B1 are essential compounds, i.e. they can only be obtained by dietary intake or by bacterial synthesis in the gut.^51^^,^^52^ Vitamin B1 has been linked to cancer cell metabolism,^53^ and a protective activity against oxidative stress in neuroblastoma cell lines has been suggested.^54^ In turn, tyrosine and phenylalanine are precursors for the synthesis of catecholamines (including the neurotransmitters dopamine, adrenaline, and noradrenaline), and alterations in urine and plasma catecholamine levels are commonly detected in children with neuroblastoma.^55^, ^56^, ^57^ Notably, all five metabolic pathways were also found enriched in the NB siblings group as compared to the patients (Fig. 3a, Wilcoxon rank-sum tests; Padj < 0.05, Table S9).

We further assessed the differences in functional potential of the gut microbiome in the NB and HC groups by computing the relative abundances of gut metabolic modules (GMMs), a manually-curated set of pathways following the general microbial metabolism in the gut,^38^ and of gut-brain modules (GBMs), another curated set of pathways focusing on the metabolism of neuroactive compounds by the gut microbiome.^37^ While no significant differences in either GMM or GBM relative abundances were detected (Wilcoxon rank-sum tests; Padj ≥ 0.05, Tables S10 and S11), when assessing differences in broader bacterial functional strategies (Methods) we found reduced saccharolytic fermentation (Fig. 3b, Wilcoxon rank-sum tests, r = 0.21, Padj = 0.03; Table S12) and increased proteolytic fermentation (r = −0.26, Padj = 0.01) in the NB group. In addition, patients with neuroblastoma displayed reduced glycoprotein degradation potential (r = 0.21, Padj = 0.03), in line with the decreased relative abundances of *Akkermansia muciniphila* (Fig. 2a), a dedicated mucin degrader in the human intestinal tract.^58^ When comparing the NB group to the NB siblings, the increased proteolytic fermentation in the patients with neuroblastoma was validated (r = −0.44, Padj = 4.10e-04; Table S13). Overall, the differences we detected highlight alterations in gut microbiome overall metabolic strategies in patients with neuroblastoma, which could in turn be linked to disease pathogenesis.

### Individuals with neuroblastoma do not show distinct mother-offspring microbiome transmission patterns

**Fig. 4:**
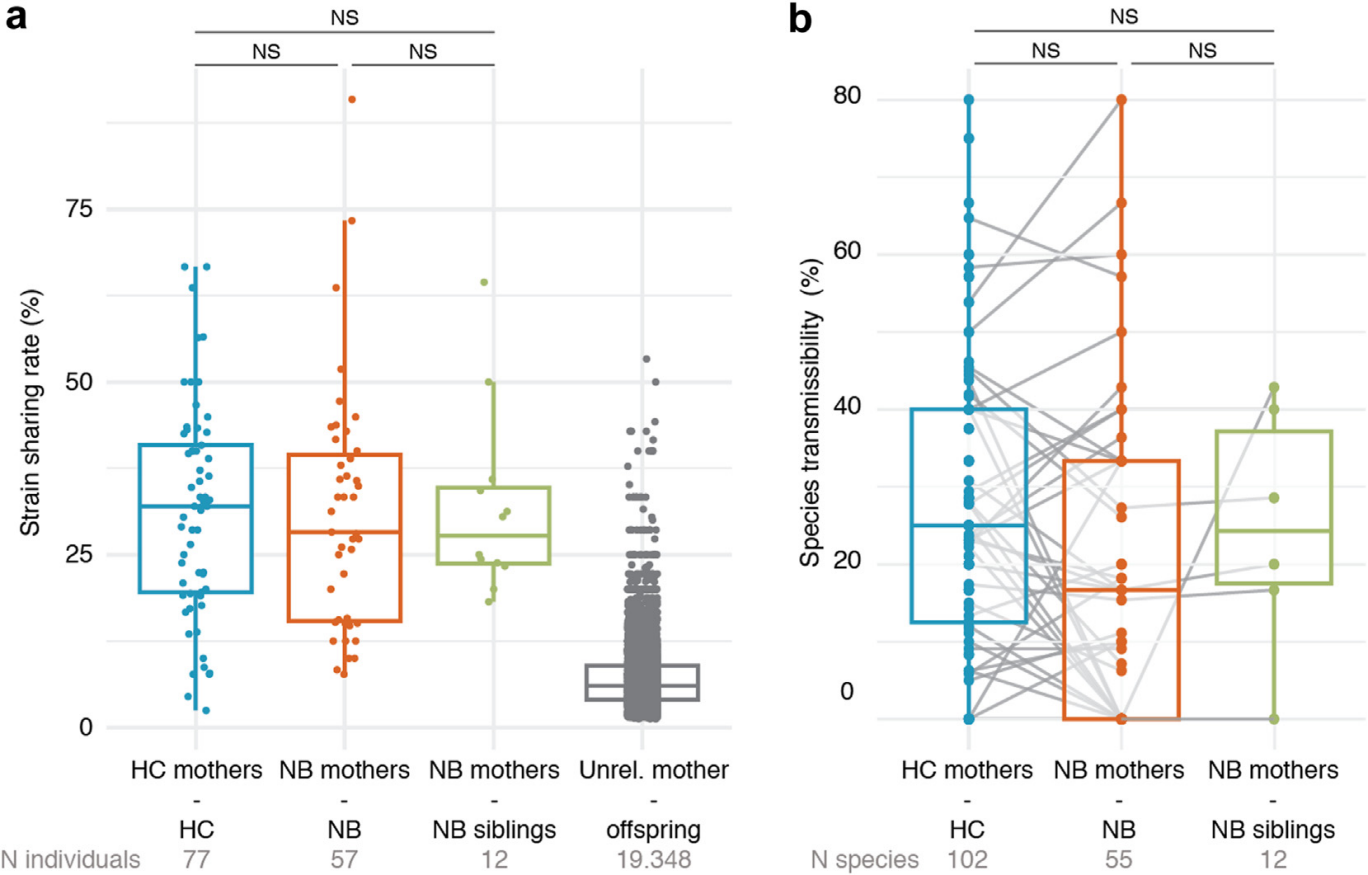
**Maternal seeding of the infant gut is not altered in patients with neuroblastoma**. **a**. No significant differences in microbial strain sharing rates (Methods) between the mothers of the HC and NB groups and the offspring are detected (Wilcoxon rank-sum tests; Padj ≥ 0.05) neither with the NB siblings group (Wilcoxon rank-sum tests; Padj ≥ 0.05). All mother-offspring pairs displayed significantly higher strain sharing rates than mothers with unrelated offspring (Table S17). For a robust calculation, person-to-person strain sharing rates were only assessed when at least 5 species were shared between two individuals (the number of pairs compared is noted under each box). **b**. No species displayed significantly different transmissibility (Methods) between mothers and offspring of the different groups (Chi2 tests on the number of pairs in which the species was transmitted and not for each species; Padj ≥ 0.05, Table S18). For a robust calculation, SGB transmissibility was only assessed on species with at least five potential strain sharing events in each category (the number of species compared is noted under each box).

The acquisition of the gut microbiome takes place majoritarily by maternal seeding in infants.^59^ The average age of diagnosis of neuroblastoma is before two years,^3^ when mother-infant microbial strain sharing rates (i.e. the number of shared strains divided by the number of species in common) is estimated to be close to 50%.^22^ As non-communicable diseases could be at least partially communicable through the microbiome,^22^^,^^27^ we here also assessed microbiome composition in mothers of the patients with neuroblastoma (NB mothers) and of healthy children (HC mothers) together with mother-infant microbiome transmission (Fig. 1a). As for the children, the two groups of mothers were balanced on the main covariates of microbiome composition (Table S2). No significant differences in beta diversity (dbRDA on Aitchison distance, P = 0.43), alpha diversity (Wilcoxon rank-sum tests, Padj ≥ 0.05 for all alpha diversity indices; Table S14), species relative abundances (Padj ≥ 0.05; Table S15), or metabolic pathways (Padj ≥ 0.05; Table S16) were detected between the two groups of mothers. However, although the overall composition of the maternal microbiome is similar, there could still be differences in either the extent of mother-offspring transmission or the frequency of transmission of disease-associated species, and we thus next assessed mother-offspring microbiome strain sharing by strain-level profiling (Methods). The extent of microbiome strain sharing between mothers and children was not significantly different in the NB (28% median strain sharing rate) and HC groups (32%) (Fig. 4a, Wilcoxon rank-sum test, r = −0.04, P = 0.64) nor when comparing the children with neuroblastoma to their healthy siblings (28%) (Fig. 4a, r = −0.01, P = 0.87). In addition, while transmissibility varied largely among the 799 species profiled at the strain level (Methods), none of them (including the species with altered relative abundances in neuroblastoma) displayed differential transmissibility between groups (Fig. 4b, Chi2 tests, Padj ≥ 0.05, Table S18). Therefore, the microbiome alterations observed in patients with neuroblastoma cannot be linked to differential maternal seeding, and they possibly arise subsequently to this first colonisation.

## Discussion

Analysis of gut microbiome composition in a cohort of patients with neuroblastoma revealed that this paediatric cancer is associated with alterations in microbiome taxonomic and functional composition that cannot be traced to differential maternal transmission of the microbiome. To our knowledge, this is the first metagenomic dataset of neuroblastoma in humans and has been set up with a sample size (N = 288 individuals) which is on par with most current investigations of the link between the gut microbiome and more common and well studied adult cancers.^12^^,^^60^^,^^61^ Importantly, while confounding can never be fully discarded in microbiome studies, in our study design we selected patients at disease onset (i.e. prior to starting any medication or treatment that could potentially modify the composition of the gut microbiome) and we carefully balanced the patient and control groups on the main known covariates of microbiome composition in both infants and adults (age, sex, stool consistency, mode of delivery, and mode of feeding), thus limiting the potential influence of confounding factors.

In our study, we found that patients with neuroblastoma are characterised by reduced gut microbial richness and decreased relative abundances of 18 species including bacteria with reported anti-inflammatory properties such as *P. dorei,* bifidobacteria, and butyrate-producers (*Roseburia, Faecalibacterium spp.*) (Fig. 2a), together with decreased potential to metabolise carbohydrates (including starch and glycogen), two amino acids (l-tyrosine and l-phenylalanine) that are precursors for the synthesis of the catecholamine neurotransmitters, and the vitamin B1 (Fig. 3a). These metabolic changes are concordant with those detected in the only study available that compared plasma metabolome composition in patients with NB to healthy children.^62^ Only one species, *Enterobacter hormaechei* (member of the *Enterobacter clocae* complex and reportedly a nosocomial pathogen), was found increased in patients, along with a more proteolytic functional profile. Such alterations were not detected in healthy siblings, which resembled the healthy nonkin group, and allowed validating most of the neuroblastoma-associated microbiome differences we found. In addition, the fact that no significant difference was detected between the mothers of the patients and of the healthy controls, and that both the overall fraction of the microbiome we assessed that mothers transmitted to offspring and the frequency of transmission of specific species were similar, suggests that neuroblastoma might arise during the neurodevelopmental changes that take place after the initial maternal seeding. Therefore, microbiome-mediated transmission of neuroblastoma seems unlikely.

After careful assessment of potentially confounding factors, we found that in such a paediatric cohort, age was a stronger covariate of interindividual microbiome variation than the disease, but with the current age-balanced study design, one can discard the microbiome alterations detected are due to the different stages of microbiome maturation in childhood.^18^ In contrast, dietary information was not available in the cohort, and nutritional intake could explain differences in both species-level and metabolic pathway composition.^63^^,^^64^ However, the fact that we largely observed the same differences when comparing the siblings to the patients and not the siblings to the control group, and that the groups of mothers did not display any significant differences in microbiome composition suggest these are not due to differential lifestyle or cultural habits of the families enrolled in the study. Next, while the current study is cross-sectional and thus neither temporal variation, causality, nor directionality of the microbiome alterations observed can yet be established, by building machine learning models that successfully discriminated patients from controls as well as from their siblings (mean AUC of 81 and 83% respectively), we found the species-level alterations hold potential as signature of the disease. Future studies will also be able to verify the generalizability of the microbiome signatures of neuroblastoma detected here across different ethnicities and lifestyles, and their robustness to different sample processing protocols.

Overall, our study showed that neuroblastoma is associated with alterations in microbiome diversity and both taxonomic and functional composition, which are not linked to maternal transmission. These findings might open the field to the possibility of developing microbiome-targeted complementary therapeutic approaches now used in other contexts (e.g. next-generation probiotics,^65^^,^^66^ including *P. dorei* and *Bifidobacterium* spp, as well as faecal microbiota transplantation^40^^,^^67^^,^^68^) to improve the symptomatology of neuroblastoma. Future studies should address the potential causal links and underlying mechanisms of these associations, the generalizability to different cohorts and ethnicities, the links between gut microbiome composition and the different stages of neuroblastoma beyond disease onset, and the effect of the most common neuroblastoma treatments (surgery, chemotherapy, radiation, and immunotherapy) on microbiome composition.

## Contributors

MV-C, MPonzoni, MVC, and NS conceptualised and designed the study. FAr, FP, AG, and LA participated in cohort recruitment and/or contributed to metagenomic data acquisition. MV-C, PM, FC, GM, FAs, AB-M and MPunčochář performed bioinformatic and statistical analyses. MV-C, MPonzoni, MVC, and NS wrote the manuscript. All authors provided critical revision of the manuscript and read and approved the final version for submission.

## Additional information

Correspondence and requests for materials should be addressed to Mireia Valles-Colomer (mireia.valles@upf.edu) and Nicola Segata (nicola.segata@unitn.it).

## Data sharing statement

Shotgun metagenomics sequencing data is available on the NCBI Sequence Read Archive database with accession PRJEB63351. Metadata are available in Table S1 and in the latest release of curatedMetagenomicData.^41^

## Code availability

All the software used in this study is publicly and freely available. The MetaPhlAn 4 package^29^ (which includes StrainPhlAn 4 and the script for strain transmission inference with the species-specific strain identity thresholds) is available at http://segatalab.cibio.unitn.it/tools/metaphlan with the open-source code at https://github.com/biobakery/MetaPhlAn and via Bioconda (https://anaconda.org/bioconda/metaphlan) and PIP (https://pypi.org/project/MetaPhlAn). HUMAnN 3.6 is available at https://github.com/biobakery/humann.

## Declaration of interests

The authors report no conflicts of interest.
