# Supplementary material for: Neuroblastoma is associated with alterations in gut microbiome composition subsequent to maternal microbial seeding
Source: eBioMedicine. 2023 Dec 16;99:104917. doi: 10.1016/j.ebiom.2023.104917 (PMC10731604; doi:10.1016/j.ebiom.2023.104917)
Supplement: Figures S1–S4 [file mmc1.pdf]

Neuroblastoma is associated with alterations in gut microbiome composition subsequent to maternal microbial seeding

Mireia Valles-Colomer[1,2^], Paolo Manghi[1*], Fabio Cumbo[1*], Giulia Masetti[1*], Federica Armanini[1], Francesco Asnicar[1], Aitor Blanco-Miguez[1], Federica Pinto[1], Michal Punčochář[1], Alberto Garaventa[3], Loredana Amoroso[3], Mirco Ponzoni[4*], Maria Valeria Corrias[4*], and Nicola Segata[1,5*^]

SUPPLEMENTARY FIGURES

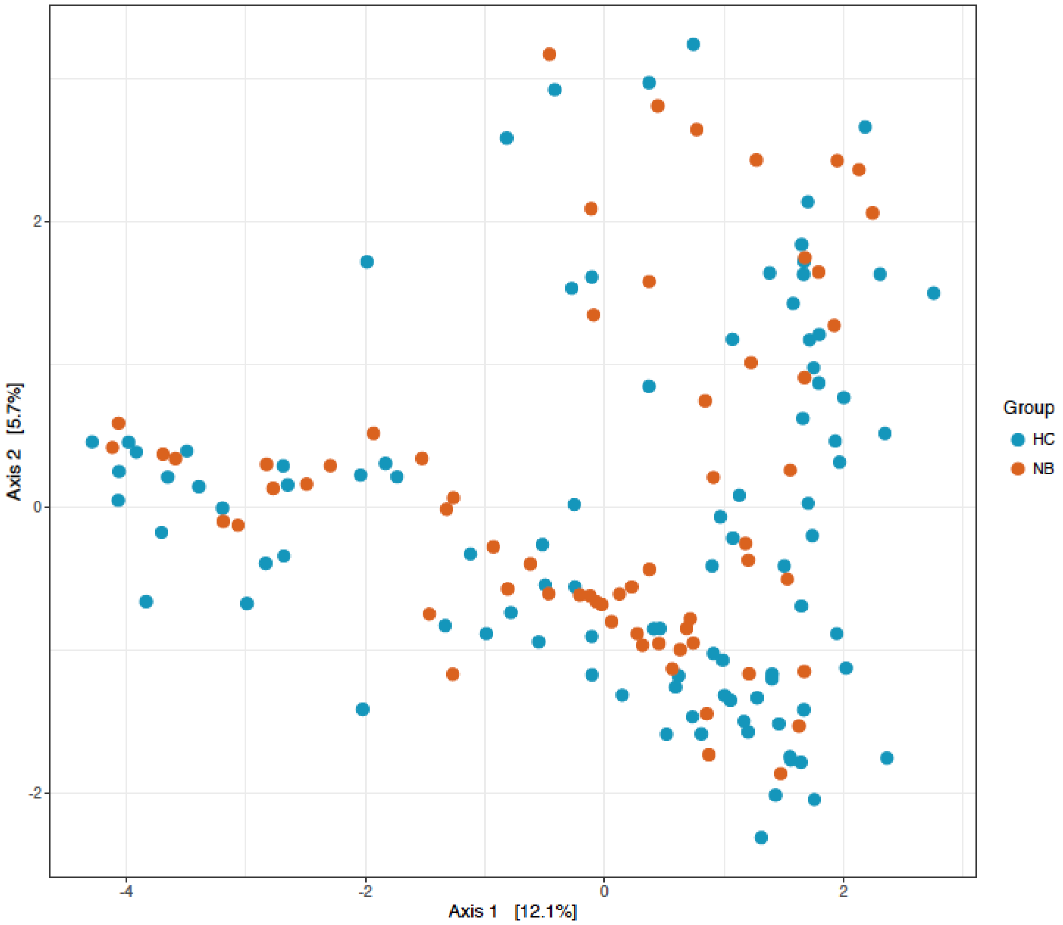

Figure S1. Species-level ordination (PCoA on Aitchison distance, N=157 samples). Samples are coloured by group (HC: healthy controls, NB: neuroblastoma), displaying a moderate but significant proportion of interindividual variation in taxonomic microbiome composition (dbRDA, adjusted R2=1.2%, P=4e-03).

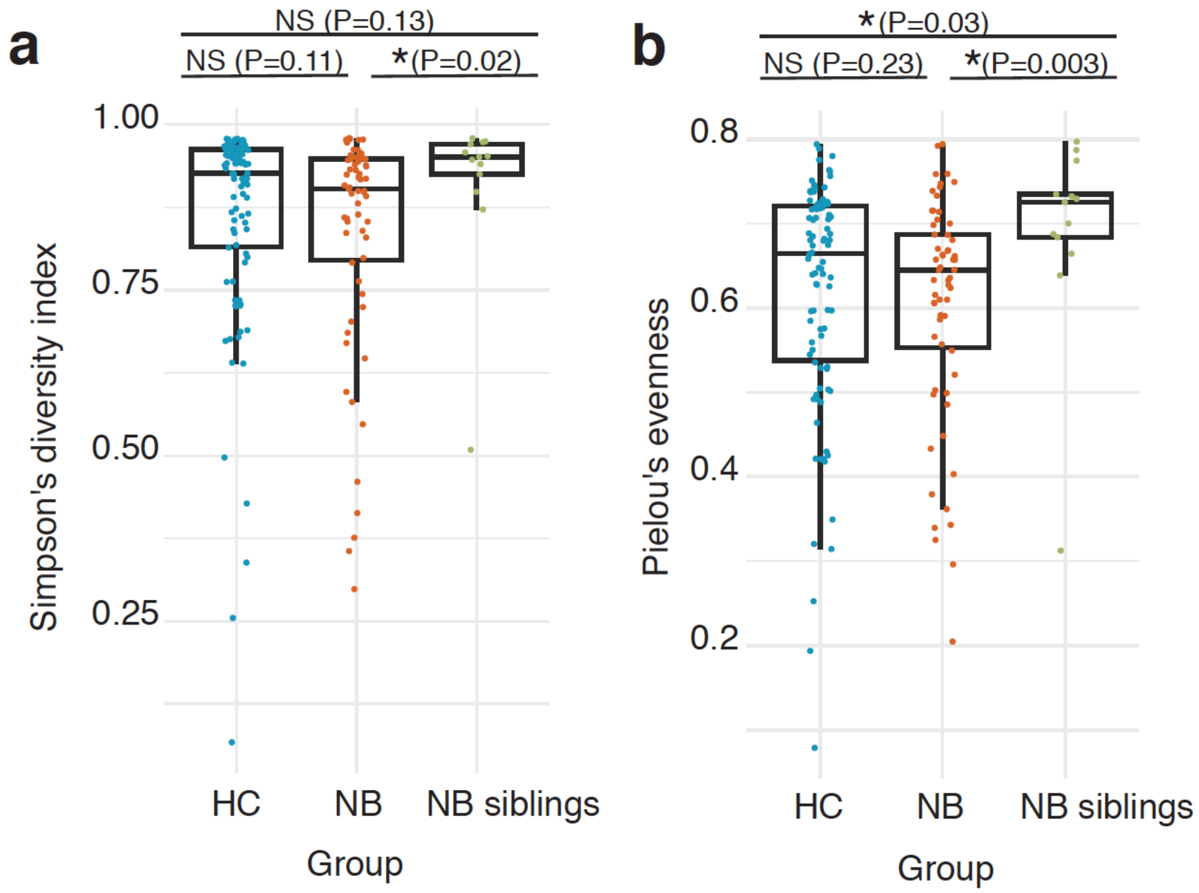

Figure S2. Species (**a**) Simpson's diversity index and (**b**) Pielou's evenness distributions in the HC (N=94), NB (N=63), and NB siblings (N=13) groups. Wilcoxon rank-sum tests (* P<0.05, NS P≥0.05; Table S4).

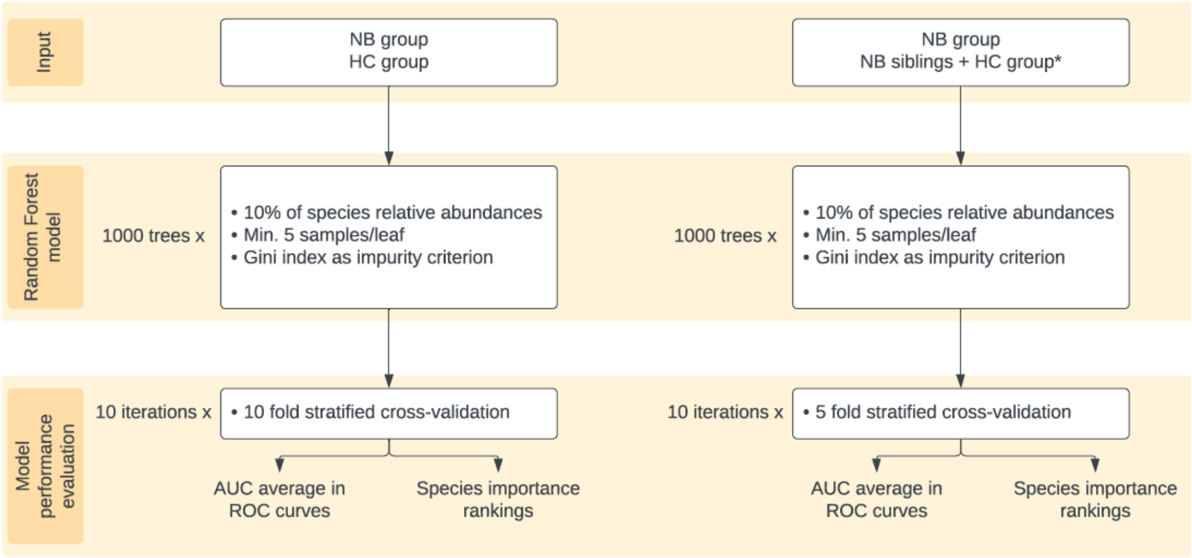

Figure S3. Workflow charts describing the procedure used to build the Random Forest models and evaluate their performance. *: in the second model, the HC group was only used to build the model, not to evaluate its performance in order to assess its applicability in the NB siblings group.

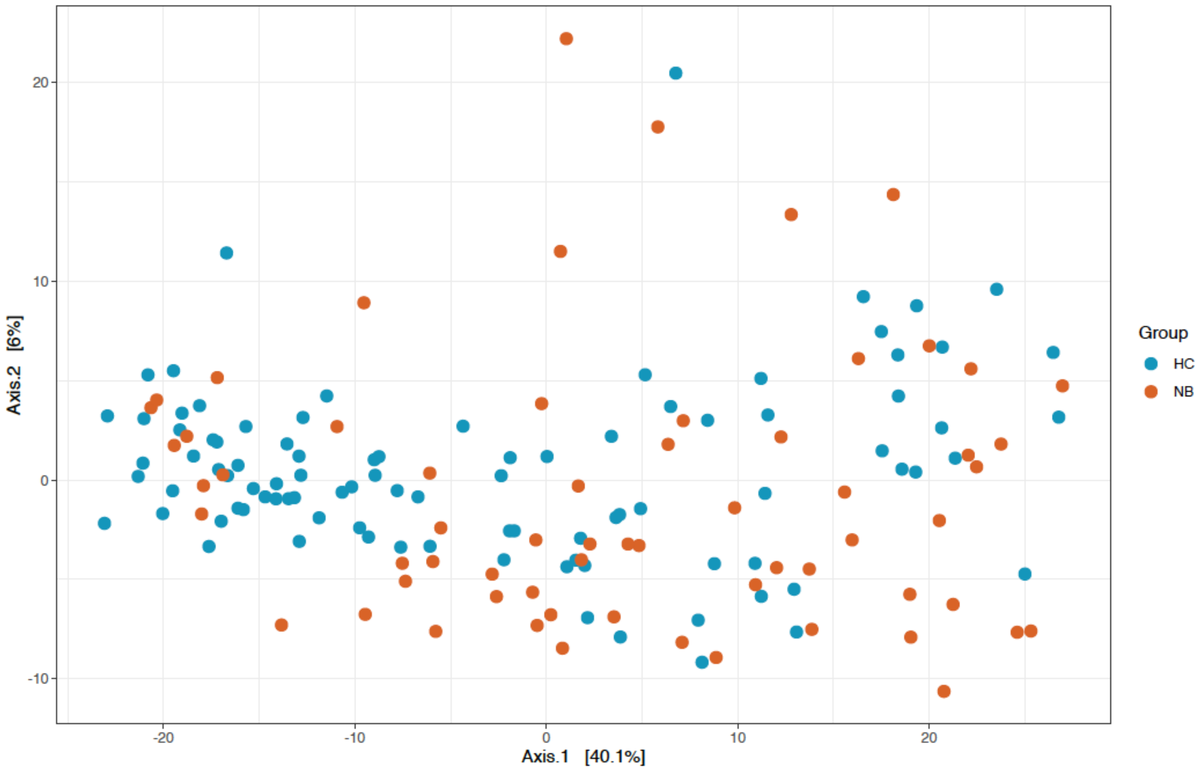

Figure S4. Functional ordination (PCoA on Aitchison distance of MetaCyc pathway composition, N=157 samples). Samples are coloured by group (HC: healthy controls, NB: neuroblastoma), displaying a moderate but significant proportion of interindividual variation in functional microbiome composition (dbRDA, adjusted R2=2.4%, P=1e-03).
